# Molecular hallmarks of ageing in amyotrophic lateral sclerosis

**DOI:** 10.1007/s00018-024-05164-9

**Published:** 2024-03-02

**Authors:** Cyril Jones Jagaraj, Sina Shadfar, Sara Assar Kashani, Sayanthooran Saravanabavan, Fabiha Farzana, Julie D. Atkin

**Affiliations:** 1 MND Research Centre, Macquarie Medical School, Faculty of Medicine, Health and Human Sciences, Macquarie University, 75 Talavera Road, Sydney, NSW 2109 Australia; 2 La Trobe Institute for Molecular Science, La Trobe University, Bundoora, Melbourne, VIC 3086 Australia

**Keywords:** Ageing, ALS, Neurodegenerative diseases, Molecular hallmarks

## Abstract

Amyotrophic lateral sclerosis (ALS) is a fatal, severely debilitating and rapidly progressing disorder affecting motor neurons in the brain, brainstem, and spinal cord. Unfortunately, there are few effective treatments, thus there remains a critical need to find novel interventions that can mitigate against its effects. Whilst the aetiology of ALS remains unclear, ageing is the major risk factor. Ageing is a slowly progressive process marked by functional decline of an organism over its lifespan. However, it remains unclear how ageing promotes the risk of ALS. At the molecular and cellular level there are specific hallmarks characteristic of normal ageing. These hallmarks are highly inter-related and overlap significantly with each other. Moreover, whilst ageing is a normal process, there are striking similarities at the molecular level between these factors and neurodegeneration in ALS. Nine ageing hallmarks were originally proposed: genomic instability, loss of telomeres, senescence, epigenetic modifications, dysregulated nutrient sensing, loss of proteostasis, mitochondrial dysfunction, stem cell exhaustion, and altered inter-cellular communication. However, these were recently (2023) expanded to include dysregulation of autophagy, inflammation and dysbiosis. Hence, given the latest updates to these hallmarks, and their close association to disease processes in ALS, a new examination of their relationship to pathophysiology is warranted. In this review, we describe possible mechanisms by which normal ageing impacts on neurodegenerative mechanisms implicated in ALS, and new therapeutic interventions that may arise from this.

## Introduction

Amyotrophic lateral sclerosis (ALS) is a relentlessly fatal and rapidly progressing disorder affecting motor neurons in the brain, brainstem, and spinal cord, resulting in gradual muscle paralysis. With a poor prognosis and severely debilitating symptoms it is important to identify the underlying mechanisms that trigger ALS. The average age of diagnosis of ALS is 55 years, and ageing is its biggest risk factor. Ageing is a slowly progressive, continuous decline in the normal function of an organism over its lifespan. It is also marked by an increased sensitivity to ageing-related diseases and increased risk of death. Importantly, the World Health Organization (WHO) estimates that the proportion of the global population over 60 years will nearly double (12% to 22%) from 2015 and 2050, implying that the incidence of age-related neurodegenerative diseases such as ALS will increase significantly in the coming decades. However, it is important to note that these estimations may be revised in the future due to the unique circumstances and challenges posed by the COVID-19 pandemic. We are currently living in the United Nations Decade of Healthy Ageing (2021–2030), a global collaboration led by the WHO, recognising the importance of ageing to health. It is also essential to note the difference between lifespan (total number of years an individual survives from birth until death) and healthspan (total number of years an individual remains healthy, without chronic disease). Thus, healthy ageing should also consider healthspan as well as lifespan.

It is well-established that changes in the morphology and function of the brain are present during ageing, involving weight and volume decreases, loss of white and grey matter, and the degeneration of neurites and synapses [1]. Whilst the effect of normal ageing on the spinal cord remains poorly studied in comparison, significant alterations have been described, including the loss of alpha motor neurons (α-MNs)[2], reminiscent of ALS. Muscle cells, like motor neurons, also display many of the classic hallmarks of ageing.

**Fig. 1:**
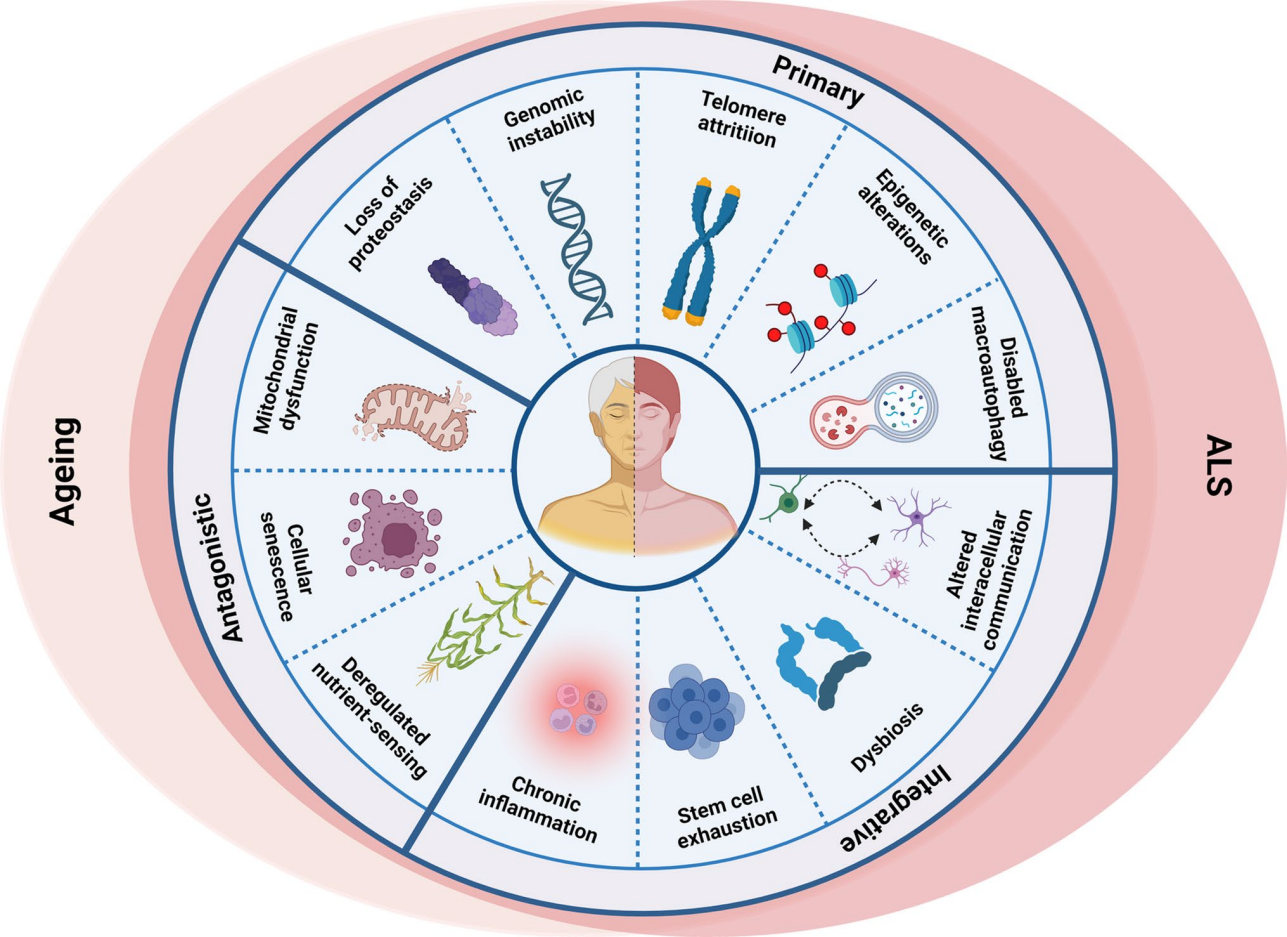
Molecular hallmarks of ageing in ALS. The molecular hallmarks of ageing denote a collection of inter-connected molecular and cellular features that are widely linked to the ageing process in diverse tissues and organisms. These mechanisms offer a framework for comprehending the intricacies of ageing. Strikingly, the molecular hallmarks of ageing exhibit notable similarities and substantially overlap with the pathophysiological mechanisms described in ALS

Whilst ageing itself is a normal process, there are striking similarities between neurodegeneration and normal ageing at the molecular and cellular level, because the specific ‘hallmarks’ associated with ageing [3] overlap significantly with pathophysiological mechanisms implicated in ALS (Fig. 1). However, it remains poorly defined how exactly ageing promotes the increased risk of ALS. The molecular and cellular hallmarks of ageing have been recently updated [3]. Hence, a new examination of the relationship between ageing and neurodegeneration in the pathophysiology of ALS is warranted and is the subject of this review.

## Ageing and neurodegeneration

Ageing occurs with time in all organisms, although it progresses at different rates within a species. The differences between individuals are due to variations in genetic makeup, environment, lifestyle, and adaptation [4, 5] and are evident on an organismal, organ, cellular and molecular level. We provide below a brief overview of the major theories of ageing.

### Theories of ageing

Whilst the factors that control human lifespan remain unclear, current theories of ageing mainly fall into two broad categories. The ‘Programmed Theory’ proposes that normal ageing follows a biological timetable (similar to that regulating childhood growth) that results in changes in expression of genes involved in cellular maintenance [6]. In contrast, the ‘Damage or Error’ theory suggests that ageing is the result of progressive damage to cells and organs over time [6]. However, currently there is no consensus on the causes of ageing in humans. Moreover, many of the cellular mechanisms implicated in ageing interact extensively and thus may act together to accelerate the underlying molecular processes.

#### The programmed theory of ageing

The programmed theory of ageing can be further divided into three subtypes. The ‘Programmed Longevity’ theory implies that ageing results from changes in gene expression, leading to age-associated deficits and a subsequent cellular senescent phenotype [7]. Secondly the ‘Endocrine Theory’ proposes that hormones act as biological clocks to control the rate of ageing [8]. Thirdly, the ‘Immunological Theory’ states that the function of the immune system is at its peak during puberty, but it declines thereafter, resulting in an increased susceptibility to inflammation [9].

#### Damage or error theory of ageing

The ‘Damage or Error’ Theory of ageing can be further divided into five subtypes. First, the ‘Wear and Tear’ theory proposes that cellular components naturally wear out over time from consistent repeated usage [6]. Second, the ‘Rate of Living’ theory states that the lifespan of an organism becomes shorter with higher rates of basal oxygen consumption [10, 11]. Third, the ‘Cross Linking Theory’ [12] proposes that proteins become cross-linked and then aggregate over time [12, 13]. The ‘Free Radical’ theory [14, 15] suggests that superoxide and other free radicals accumulate and damage cellular components (nucleic acids, lipids, sugars, and proteins) during ageing [15]. Whilst antioxidants counteract this to some extent, eventually this becomes ineffective during normal ageing [15]. Finally, the ‘Somatic DNA Damage’ theory proposes that DNA damage occurs continuously in cells. Whilst these lesions are initially repaired, increasing damage over time results in mutations, which impair genome integrity and thus cellular function. Damage to both nuclear and mitochondrial DNA is implicated in this process [16].

### Genetics and ageing in ALS

It remains unclear how ageing increases the risk of ALS, but it probably involves a combination of genetic, environmental, and age-related factors [17]. Whilst most (~ 90%) ALS cases arise sporadically, the remaining proportion are familial, and can provide insights into the underlying pathophysiology [17]. Hexanucleotide repeat expansions (GGGGCC) in the first intron of chromosome 9 open reading frame 72 (*C9ORF72*) gene are the most common genetic cause of both familial (~ 40%) and sporadic ALS (~ 8–10%), as well as the related condition, frontotemporal dementia (FTD), both sporadic (~ 5–10%) and familial forms (~ 25–30%) [18]. FTD primarily affects the frontal and temporal lobes of brain and patients exhibit a combination of cognitive, behavioural, and/or motor symptoms, although these can vary widely [19]. Some individuals with FTD may also develop symptoms of ALS and vice versa, referred to as 'ALS-FTD' [20]. Hence, there is significant genetic and pathological overlap between ALS and FTD. Three main mechanisms are implicated in neurodegeneration induced by hexanucleotide *C9ORF72* mutations; production of toxic RNA, non-AUG translation (RAN) to produce dipeptide repeat proteins (DPRs) and haploinsufficiency due to lack of C9ORF72 protein [21].

Mutations in the genes encoding superoxide dismutase 1 (SOD1) and TAR DNA-binding protein 43 (TDP-43) cause another ~ 20% and ~ 4% cases respectively of familial ALS cases [22–24]. TDP-43 is an RNA/DNA binding protein normally located primarily in the nucleus. However, the presence of pathological forms of TDP-43, involving its truncation, abnormal aggregation, and mislocalization to the cytoplasm, are the characteristic hallmark of almost all (~ 97%) ALS cases [25]. Fused in Sarcoma (FUS) is another RNA-binding protein with structural and functional similarities to TDP-43 and mutations in FUS also cause ~ 4% cases [26]. Over 30 other genes have been linked to familial ALS, though each account for a smaller proportion of cases. These genes include *CCNF, CHCHD10, ATXN2, KIF5A, hnRNPA2/B1, UBQLN2, TBK1. OPTN, PRPH, NEK1, VCP,* and *PFN1*, among others [17, 27, 28].

Whilst ALS involves the degeneration and death of motor neurons, glial cells, which provide important supportive roles to neurons, also contribute to pathophysiology via non-cell autonomous mechanisms. Astrocytes regulate blood flow within the CNS, recycle neurotransmitters, and form the blood brain barrier. Microglia function in phagocytosis, the immune response, neuroinflammation, and immune surveillance and activation. Thus they act as the immune cells of the CNS [29]. Oligodendrocytes myelinate neuronal axons within the CNS to facilitate synaptic transmission and provide metabolic support to neurons, and Schwann cells myelinate neuronal axons in the peripheral nervous system (PNS). The latter cells also perform important roles in maintaining the function of the neuromuscular junction (NMJ) [30].

The clinical manifestations in ALS are driven by loss of voluntary muscle function, facilitated normally by motor neurons at the NMJ [31, 32]. Previously, ALS was considered to affect motor neurons primarily, and the involvement of skeletal muscle was thought to be a secondary consequence. However, the role of muscle in the pathogenesis of ALS is gaining increasing recognition (reviewed recently [33]).

## Molecular hallmarks of ageing in ALS

**Fig. 2:**
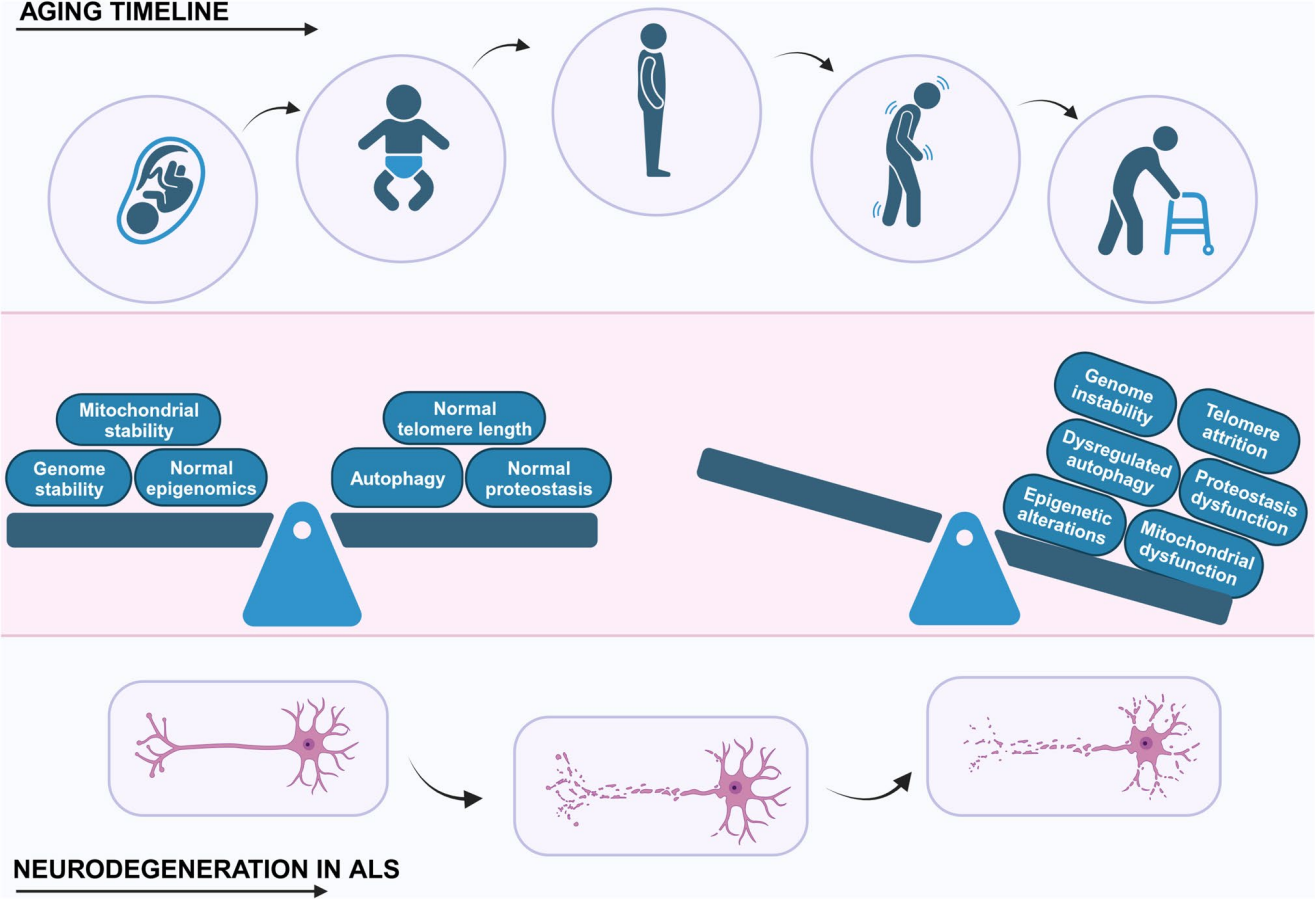
Primary drivers of ageing and ALS. Ageing and neurodegeneration in ALS are complex processes influenced by a combination of genetic, environmental, and cellular factors. Whilst the precise causes of both ageing and ALS are not fully understood, genomic instability, telomere attrition, epigenetic alterations, proteostasis dysfunction, dysregulated autophagy and mitochondrial dysfunction are thought to be primary drivers

The molecular and cellular hallmarks of ageing are defined by specific criteria [3]; (a) a hallmark should alter in a time-dependent fashion during the ageing process, (b) it should be enhanced by experimental acceleration of ageing, and (c) modulating the hallmark should inhibit, halt or even reverse ageing. Nine ageing hallmarks were originally proposed (in 2013) [3]: genomic instability, loss of telomeres, senescence, epigenetic modifications, dysregulated nutrient sensing, loss of proteostasis, mitochondrial dysfunction, stem cell exhaustion, and altered intercellular communication. However, these hallmarks were recently (2023) [3] expanded to include dysregulation of autophagy, inflammation and dysbiosis. It is important to note however that these twelve age-related hallmarks overlap significantly and are highly inter-related, with much crosstalk between these pathways (Fig. 1). Several are implicated as ‘primary’ hallmarks and drivers of the ageing process [3], including genomic instability, telomere dysfunction, epigenetic dysregulation, and proteostasis dysregulation (Fig. 2). In contrast, the ‘antagonistic’ hallmarks refer to cellular reactions to damage, including nutrient-sensing, mitochondrial dysfunction, and senescence. Finally, the ‘integrative’ hallmarks reflect the lack of ability of the cell to cope with the age-associated damage, involving defects in inter-cellular communication, stem cell exhaustion and dysbiosis. Below, we detail each of the twelve hallmarks, and how these relate to mechanisms of neurodegeneration in ALS.

### Primary hallmarks of ageing and how they relate to ALS

#### Genomic instability

Genomic instability refers to the high frequency of mutations within the genome [34]. This can result from both exogenous and endogenous sources, such as environmental agents and DNA replication errors, respectively. The DNA damage response (DDR) refers to the signalling pathways that normally detect and repair DNA damage, and the efficiency of DNA repair decreases during ageing [3]. Genomic instability results from either alterations in the nuclear architecture, damage to nuclear and/or mitochondrial DNA, and defective DNA repair mechanisms [35]. However, whilst genomic instability increases significantly with ageing, direct evidence showing that it modulates ageing specially is lacking.

##### Nuclear architecture alterations

The architecture of the nucleus maintains multiple aspects of genome stability. This primarily involves the nuclear lamina, a filamentous scaffold mesh underneath the nuclear envelope that tethers proteins and chromatin. Nuclear lamin proteins are its main constituents, and they are strongly associated with ageing and genome stability. Importantly, mutations in the genes encoding several of these proteins cause accelerated ageing disorders such as Hutchinson–Gilford progeria syndrome (HGPS, or progeria) [36, 37], which results from an abnormal truncated form of Lamin A (progerin), that also accumulates normally with age [38]. Dysregulation of Lamin B1 disrupts the shelterin complex and drives telomere instability in human cells [39].

Defects to the nucleus and impairment of nucleocytoplasmic transport are well-described in ALS. Nuclear pore pathology is detected in brains of sporadic ALS, TDP-43 and C9ORF72 patients [40]. Pathological forms of TDP-43 disrupt the nuclear architecture and nuclear pore complexes in ALS [40]. ALS-associated variant FUS^R521G^ interacts with nucleoporins, which form the nuclear pore complex, and disrupts nucleocytoplasmic transport [41]. The C9ORF72 RNA and DPRs also interact with and disrupt various components of the nuclear transport machinery such as nuclear transport receptors, Ran GTPase, nucleoporins and nuclear envelope proteins [42]. However, nuclear morphology is unaltered in C9ORF72 ALS/FTD [43]. Loss of the nucleoporin NUP50 has been implicated as a risk factor for ALS [44]. Mutations in loss of never-in-mitosis A (NIMA)-related kinase-1 (NEK1) in induced pluripotent stem cell (iPSC) derived motor neurons also disrupt the nuclear architecture and import of proteins [45].

##### Damage to nuclear DNA

Cells are highly prone to DNA damage and insults arise at a rate of tens of thousands per day per cell [34]. Somatic mutations normally accumulate over time and the rate of formation is inversely correlated with lifespan [3]. During normal ageing, the efficiency of DNA repair mechanisms declines, resulting in the accumulation of DNA damage [35]. Furthermore, mutations in several DNA repair proteins cause several human progeroid disorders, directly linking DNA repair deficiencies to ageing. Double-stranded DNA breaks (DSBs) are the most toxic type of damage, which in neurons are repaired primarily by the error-prone non-homologous end-joining (NHEJ) mechanism. Neurons are also prone to oxidative DNA damage, which is repaired by base excision repair (BER) [46].

There is now extensive evidence for DNA damage in the pathophysiology of ALS. Several proteins central to ALS, including C9ORF72, FUS, TDP-43, SOD1, NEK1, C21orf2, senataxin, and valosin containing protein 1 (VCP), are known to function in DNA repair [47]. We and others have shown that TDP-43 is recruited to γH2AX foci where it functions in NHEJ [48], interacts with Ku 70 and is implicated in the repair of R loops [49, 50]. FUS interacts with histone deacetylase 1 (HDAC1) to repair DSBs [51] and it also functions in BER by mediating PARP1-dependent recruitment of XRCC1/DNA Ligase IIIα (LigIII). C21orf72 interacts with NEK1 and is thought to be involved in DSB repair [52–54]. VCP and senataxin are also involved in the maintenance of genomic integrity by facilitating transcription, DNA replication and the DDR [55, 56].

DNA damage is also induced by pathological forms of the same proteins in ALS [57]. ALS-mutant TDP-43 displays impaired activity in NHEJ, which disrupts R-loop homeostasis and induces TDP-43 pathology [48, 58]. TDP-43 pathology is associated with genome instability, encompassing splicing changes, somatic mutations, and gene fusions [59]. Loss of TDP-43 in the nucleus correlates with increased accumulation of DSBs [60, 61]. Similarly, ALS-associated mutant FUS^R521C^ induces DNA damage and RNA splicing defects [62]. Loss of nuclear FUS impairs DNA nick ligation by inhibiting recruitment of XRCC1/LigIII [63], inducing aggregate formation and neurodegeneration [64]. In addition, ALS-associated variants of other proteins implicated in ALS also induce DNA damage. DNA repair genes are activated in response to DNA damage caused by SOD1^G93A^ mutations in iPSC-derived motor neurons [65]. Hexanucleotide mutations in *C9ORF72* induce DNA damage in neuronal cells, and motor neurons of ALS patients [66]. This has been associated with deficiencies in DSB and R loop repair and H2A ubiquitylation [67]. The C9ORF72 DPRs poly-glycine arginine (poly-GA) and poly-proline-arginine (poly-PA) induce DSBs, and phosphorylation of ataxia telangiectasia mutated (pATM) [68]. There is also evidence linking DNA repair defects to motor neuron loss. *Ercc1*^Δ/−^ mice lacking DNA repair mechanisms nucleotide excision repair (NER), inter-strand crosslink repair, NHEJ and homologous recombination (HR) display aberrant motor neuron loss, microglia and astrocyte activation, Golgi apparatus dysfunction, genotoxic stress and NMJ pathology [69]. However, neither TDP-43 nor FUS pathology were detected in motor neurons in these mice, indicating that loss of *Ercc1* alone is enough to induce ALS-related pathology [69, 70]. Together these data imply there is a strong correlation between ALS and DNA damage, raising the possibility that normal ageing increases genomic instability and thus the risk of neurodegeneration. However, this has not been shown directly.

##### Damage to mitochondrial DNA

Mitochondrial DNA (mtDNA) is particularly vulnerable to age-associated somatic mutations because of its proximity to oxidative phosphorylation sites and lack of protection by histones [71]. It accumulates oxidative damage in an age-dependent manner [71]. Furthermore, whilst mtDNA repair mechanisms are not as well-studied as those of nuclear DNA, they appear to be less efficient [71].

Mutations in mDNA and increased oxidative stress are implicated in both ageing and the development of ALS [72, 73]. Both wildtype TDP-43 and mutant TDP-43^Q331K^ localise to mitochondria and trigger the release of mtDNA through the mitochondrial permeability transition pore [74]. The mtDNA accumulation then activates the cGAS/STING pathway, inducing neuroinflammation and neurodegeneration [74]. Cytoplasmic mtDNA is also present in spinal cords of ALS patients and iPSC-derived motor neurons [74]. Therefore, together these studies imply that damage to mtDNA is present in ALS, although this is not well-characterised.

#### Telomere attrition

Telomeres are non-coding repetitive DNA sequences (TTAGGG)_n_ found at the distal ends of chromosomes that protect the integrity of the genome during replication. During normal ageing, the length of telomeres decreases, and rodents with short or long telomeres display inhibition or extension of lifespan, respectively [3]. Telomere shortening is thus one of the major features of ageing that is implicated in many age-related diseases [75]. Telomerase reverse transcriptase (TERT) prevents telomere shortening by maintaining telomere length [75], and whilst telomere shortening induces genomic instability and DNA damage, it is recognised as a separate hallmark of ageing [3].

Dysregulation in the length of telomeres has also been described in ALS. Knockout of telomerase leads to telomere shortening and an accelerated ALS phenotype in the SOD1^G93A^ mice model [76]. In addition, age-dependent telomere shortening was detected in iPSC motor neurons from C9ORF72 patients [77]. However, a recent whole genome sequencing study concluded that longer telomeres are a risk factor for ALS and worsen prognosis, including in the brain [78]. Similarly, longer telomere length is associated with FTD [79]. Hence, it is possible that maintaining a balanced telomere length is essential in ALS and that alterations in telomere length, both lengthening and shortening, are both relevant to neurodegeneration. In contrast, genome wide association studies found no association between telomere length and ALS in leukocytes, implying that telomere length is cell-type specific [80]. Thus, these contrasting findings imply that more studies are required to characterise telomere length and activity in ALS.

#### Epigenetic alterations

Epigenetics refers to heritable changes in the regulation of gene expression independent of the DNA sequence. Multiple epigenetic modifications are known to alter during ageing [81], including DNA methylation, histone acetylation, chromatin remodelling and regulation of non-coding RNAs [81]. These alterations affect DNA replication and repair, gene transcription and silencing, cell division, and maintenance of telomere length [81]. DNA methylation on cytosine is one the most studied epigenetic modifications.

Chromatin, containing both genomic DNA and histones, regulates accessibility of the transcription machinery and thus gene expression. During ageing, chromatin alterations occur, including structural remodelling and changes in chromatin architecture, loss of histones and histone post-translational modifications. Histone acetylation is regulated by histone acetyltransferases (HATs) and histone deacetylases (HDACs) [82, 83]. Decreased global histone acetylation results in dysregulated metabolic gene expression and metabolic homeostasis [82]. Hyper-or hypo-acetylation of histones is regulated by HAT/HDAC homeostasis, and imbalance in this process induces defects in the integrated stress response and DNA repair mechanisms [84]. HDAC inhibitors have been implicated as a therapeutic strategy to prevent ageing [85].

There is increasing evidence for a role for epigenetic modifications in the pathogenesis of ALS, particularly in relation to the C9ORF72 repeat expansion [86]. Increased methylation of a CpG island near the GGGGCC repeat in the C9ORF72 promoter decreases C9ORF72 protein expression [87]. Furthermore, age-accelerated DNA methylation in the CpG-island 5′ is associated with more severe disease phenotype, early onset, and short disease duration in C9ORF72 patients [88]. Histones H3 and H4 undergo hyper-methylation of the promoter CpG-island [89] in ALS and FTD patients [90–93]. Hypermethylation also inhibits the formation of RNA foci and DPR aggregation in ALS [94]. Less nuclear 5-methyl cytosine (5mC) and 5hmC methylation was detected in lower motor neurons displaying TDP-43 pathology compared to those lacking pathology [95]. In addition, iPSC-derived motor neurons from ALS-associated *FUS* variants express more DNA methyltransferases and display more methylation in the *FUS* promoter region [96]. Studies in SOD1^G93A^ mice also identified aberrant DNA and RNA methylation (increased or decreased) in spinal cords and skeletal muscles compared to control mice [97].

Epigenetic alterations to chromatin are also described in ALS. A chromatin remodelling complex, neuronal Brahma-related gene 1 (Brg1)-associated factor complex (nBAF), which in neurons regulates differentiation, dendritic expansion, and synaptic function, was lacking in cultured motor neurons expressing ALS-associated FUS^R521G^ or TDP-43^G348C^ [98]. Wildtype TDP-43 expression also disrupts chromatin dynamics due to impaired functioning of the chromatin remodelling enzyme CHD2 in *Drosphilia* [99]. HAT/HDAC homeostasis is altered in the brain and spinal cord of FUS-ALS patients [84]. HDAC inhibition using ACY-738 restores global histone acetylation, improves survival, and reduces metabolic abnormalities in a mouse model overexpressing wildtype FUS [100]. HDAC inhibitors have been examined extensively in ALS models (SOD1^G93A^ mice, FUS and C9ORF72 mice models (detailed further in the "Therapeutic interventions for ageing and ageing-related diseases" section) [85, 101].

#### Loss of proteostasis

Protein homeostasis, or 'proteostasis', refers to the dynamic network of processes that regulate the protein synthesis, folding, trafficking and degradation machinery [102]. Proteostasis depends on the proper functioning of molecular chaperones, autophagy, the ubiquitin proteasome system (UPS), and endoplasmic reticulum (ER)-associated degradation (ERAD). Loss of proteostasis occurs if these protein quality control mechanisms fail and this can result in the accumulation of misfolded or aggregated proteins [103]. During normal ageing, the efficiency of proteostasis declines, and the consequent accumulation of damaged and aggregated misfolded proteins is a key hallmark of ageing and neurodegeneration. Lipofuscin aggregates—granules composed of misfolded proteins and lipids as a by-product of lysosomal digestion—also accumulate in motor neurons during normal ageing [104–106]. Proteins can also become post-translationally modified during ageing by oxidative damage from reactive oxygen species (ROS) or sugars, and the later modification results in the formation of advanced glycation end products (AGEs)[107]. The rate of protein translation decreases with age, and slowed translation elongation induces protein misfolding and ageing [108]. Proteostasis collapse refers to the breakdown or failure of the cellular machinery responsible for maintaining protein homeostasis and it is implicated as an important driver of cellular ageing in humans [103, 109].

The expression of protein chaperones such as the heat shock proteins (HSPs) decreases with ageing [110], implying that protein folding becomes impaired with increasing age. Administering recombinant human HSP70 to mice delays senescence, enhances proteasome activity and cognitive functions, reduces brain lipofuscin levels, and extends lifespan [111]. Feeding young fruit flies with AGEs and lipofuscin inhibits the UPS, which accelerates ageing and reduces lifespan [112]. Similarly, another chaperone, the oxidoreductase protein disulphide isomerase (PDI) is protective against cellular ageing in several models including replicative senescent human mesenchymal stem cells (RS hMSCs), HGPS hMSCs, Werner syndrome (WS) hMSCs and human primary hMSCs [113]. In addition, stabilising dysfunctional proteostasis using the chemical chaperone 4‐phenyl butyrate (PBA) improves cognitive behaviour and inhibits ageing [114].

The pathological ALS hallmark of misfolded protein aggregates  strongly implicates proteostasis defects in pathophysiology [102]. Dysregulation of most proteostasis and protein quality control mechanisms are also well-described in ALS, including defects in autophagy, the UPS, ER-Golgi transport and ERAD [102]. Numerous molecular chaperones are also dysregulated in ALS including PDI proteins and HSPs [102]. PDI proteins have also been linked to ALS as a protective mechanism and as a genetic risk factor [115–118].

The formation of stress granules (SGs) is increasingly recognised in the maintenance of proteostasis [119]. SGs are cytoplasmic membrane-less organelles (also known as biomolecular condensates) composed of protein and RNA [120–123]. Functionally, they are implicated in the storage of biomolecules and as mRNA triage locations to regulate translation and the stability of mRNA [124, 125]. The formation of SGs is regulated by liquid phase separation (LLPS), the process by which proteins and nucleic acids in solution separate into liquid droplets (similar to droplets of oil forming in water) [126]. SGs assemble and disassemble in response to exogenous or environmental conditions, thus promoting survival during cellular stress [127]. LLPS is driven by proteins with intrinsically disordered domains, which includes misfolded proteins associated with ALS, including TDP-43 and FUS [128]. Recent studies have shown that SGs sequester misfolded proteins, preventing them from building up in the nucleus or cytoplasm, thus maintaining proteostasis [119]. However abnormal SGs disrupt proteostasis and during normal ageing defects in regulating the normal assembly/disassembly and dynamics of SGs is related to loss of proteostasis [33].

SGs are present in the pathological aggregates in ALS. Moreover, they are implicated in the formation of misfolded protein inclusions via nucleation of these aggregates [129]. TDP-43 localises in SGs in the presence of ER stress, oxidative stress, mitochondrial stress, osmotic stress, and inhibition of the proteasome [49, 130–134]. ALS-associated variant SOD1^G93A^ colocalizes with SGs, unlike wildtype SOD1 [135, 136]. Co-localization of TDP-43 aggregates and SG markers has been detected in ALS patient tissues [120, 130, 137, 138], although cellular studies could not detect co-localization between mutant TDP-43 ^A315T, M337V^ and SGs under stress conditions [139, 140]. Similarly, colocalization between ALS-mutant FUS ^R495X^ and SGs has been reported in cell lines, primary neurons, human tissues [137, 141–145].

#### Dysregulated macroautophagy

Autophagy is a catabolic process responsible for the degradation and recycling of cellular components. Macroautophagy is the major type of autophagy, which involves the formation of double-membraned vesicles, or autophagosomes. Dysregulation of macroautophagy is well-described in ageing and was recently categorised as a separate hallmark from proteostasis, because organelles and non-protein cellular components are also subject to macroautophagy [3]. Expression of autophagy-related genes, including *ATG5, ATG7,* and *OPTN*, are known to decline with age [146, 147]. This results in the accumulation of protein aggregates and dysfunctional organelles during ageing [146]. In addition, stimulation or activation of autophagy increases healthspan and lifespan in humans and model organisms [146]. Autophagy is also reduced in muscle samples obtained from elderly patients [148]. Knocking out autophagy-related gene 7 (*ATG7*) in mice leads to increased muscle atrophy, muscular inflammation, abnormal structure, and reduced lifespan [148].

Dysregulated macroautophagy is implicated in neurodegeneration in ALS [149] and ALS-associated variants in *C9ORF72, SOD1, TARDBP, TBK1, FUS, FIG4, OPTN, UBLN2, SQSTM1, CHMP2B, ALS2* dysregulate macroautophagy [150]. When autophagy is inhibited genetically or pharmacologically, ageing is accelerated and motor neuron toxicity is enhanced in ALS [146, 151]. Autophagy also plays a vital role in clearing protein aggregates associated with neurodegeneration in ALS [152]. Increased activation of autophagy proteins is detected in SOD1^G93A^ transgenic mice [153]. Similarly, progesterone is neuroprotective through activation of autophagy in SOD1^G93A^ mice [154]. C9ORF72 itself interacts with Rab1a and Unc-51-like kinase 1 (ULK1) complex to initiate autophagy via the formation of autophagosomes [155] and loss of C9ORF72 impairs autophagy [156, 157]. C9ORF72 DPRs co-localise with including p62-positive inclusions, suggesting that DPRs are targeted for clearance by the UPS and/or autophagy [158]. TDP-43^A315T^ mutation activates ER stress and induces autophagy to clearance misfolded protein aggregates [159].

### Antagonistic hallmarks of ageing in ALS

#### Cellular senescence

Senescence is implicated as an important characteristic and driver of the ageing process. Many studies have shown that senescence regulates age-associated phenotypes and is present in age-related diseases [160, 161]. Senescent cells were previously considered to be harmful because their elimination extends the lifespan of mice [162]. However, more recent studies in liver have reported that senescent cells positively impact on healthy ageing and lifespan and may have important functional roles in ageing [163]. In cycling cells, senescence is characterised by a state of eternal cell cycle arrest although  they remain metabolically active [164]. DNA damage in the nucleus (mainly in the form of DSBs) and telomere shortening are key features of senescence [165–169]. The senescence-associated DDR involves ATR, ATM, and p53, which induces activation of cyclin-dependent kinase inhibitors p16, p21, and p27 and hyperphosphorylation of retinoblastoma protein (Rb), which results in withdrawal from the cell cycle [170]. Senescence is induced following diverse endogenous and exogenous stimulii, including oxidative stress, neuroinflammation, oncogenic activation, inactivation of tumour suppressor genes and mitochondrial dysfunction [168, 171]. During senescence, cells undergo several phenotypic modifications, including profound chromatin and secretome changes, and tumour-suppressor activation [172]. The senescence-associated secretory phenotype (SASP) is a prominent feature of senescence that induces inflammation via accumulation of pro-inflammatory cytokines, chemokines and growth factors [173]. Consequently, senescent cells can induce significant alterations in the cellular microenvironment through SASP, which can worsen inflammation [174]. Microglia in the white matter are thought to be the primary cell type undergoing senescence in the CNS during ageing [175].

As neurons are post-mitotic, they do not undergo classic ‘replicative’ senescence, so this mechanism was originally thought to be restricted to dividing cells. However, neurons express senescence markers, SASP is present in the ageing brain, and recent findings have revealed that neurons undergo a similar process as senescence in response to stress (‘stress-induced premature senescence’) [170, 176–179]. Hence, senescence in normal ageing neurons may compromise viability and increase their susceptibility to additional insults [180]. However, our understanding of senescence in neurons remains limited [181].

Senescence has been described in ALS, although this has been detected predominately in glial cells. In lumbar spinal cords of symptomatic SOD1^G93A^ rats, microglia with characteristics of senescence were detected [174]. Senescence markers, including loss of nuclear lamin B1 expression and significantly increased p16INK4a, p53, matrix metalloproteinase-1 (MMP-1) were present compared to non-transgenic or asymptomatic transgenic rats [174]. Interestingly, other cell types in the degenerating lumbar spinal cord, including ChAT-positive motor neurons and GFAP-expressing astrocytes, also exhibited nuclear p16INK4a staining. Similarly, in astrocytes generated from iPSCs of individuals with sporadic ALS and ALS-C9ORF72 patients, there was a significant rise in expression of senescence markers [182]. The brains of ALS patients also display elevated numbers of senescent astrocytes [183].

Satellite cells are skeletal muscle adult stem cells that reside between muscles fibres and basement membranes and self-replicate and/or differentiate to new form new muscle fibres following injury [184]. Senescence has been reported in these cells in geriatric mice, resulting in halted muscle fibre regeneration [185]. B lymphoma Mo-MLV insertion region 1 homolog (Bmi1) knockdown results in senescence-like phenotypes in young satellite cells [185]. Protein arginine methyltransferase 7 (PRMT7) [186] is associated with muscle regeneration capacity and its expression decreases in an age-dependant manner [187]. Decreased skeletal muscle mass, impaired satellite cell regeneration and premature senescence, were detected in PRMT7 knockout mice [186]. Together these findings lend support to the idea that senescence plays a role in the development of ALS, although this is not well-characterised [182].

#### Mitochondrial dysfunction

Mitochondria are multi-functional organelles that have long been associated with ageing. They provide the primary sources of cellular energy, and also regulate innate immunity, inflammation and apoptosis [188]. During ageing, mitochondrial functions become impaired by defects in membrane potential, decreased respiratory capacity, increased free radical production, reduced turnover, and dynamics, as well as accumulation of mutations in mtDNA [73, 188].

Mitochondrial dysfunction is widely described in ALS [73, 188, 189]. Deficiencies in mitochondrial respiratory chain complex 1 are present in motor neurons obtained from lumbar spinal cord sections from sporadic ALS patients [106, 190]. Decreased mitochondrial membrane potential is present in C9ORF72 and mutant TDP-43^M337V^ human iPSC-derived motor neurons [191] and fibroblasts [192]. C9ORF72 haploinsufficiency impairs mitochondrial bioenergetics and function, and expression of electron transport chain complexes [193, 194]. Overexpression of C9ORF72 DPRs (particularly poly-GR) induces mitochondrial DNA damage, disrupts mitochondrial membrane potential, and increases ROS production [195]. Poly-GR binds to mitochondrial ATP synthase Atp5a1, inducing defects in mitochondrial structure and morphology [196]. Abnormal accumulation of mitochondria is present in spinal cord motor neurons of mutant TDP-43^A315T^ and SOD1^G93A^ transgenic mice [197] and mitochondria dysfunction and transport abnormalities are present in cells expressing ALS mutant TDP-43^Q331K, M337V^ [198–200] and mutant SOD1^G93A, G85R^ [201]. Mutations in UBQLN2^P497S^ [202] and FUS^R514G^ also induce mitochondrial abnormalities [203]. ALS-linked oxidised SOD1 triggers mitochondrial dysfunction and cellular senescence, which further accelerates ageing, providing a more direct link between oxidative stress, ALS and ageing[204]. Together, these findings suggest that mitochondrial dysfunction is closely associated with the major ALS pathological proteins.

#### Dysregulated nutrient sensing

During ageing, there is a decline in key metabolic signaling pathways relevant to ageing and neurodegeneration [210], involving the adrenergic, dopamine, insulin/insulin-like growth factor 1 (IGF1), AMP-activated protein kinase (AMPK), sirtuin (SIRT) and mTOR pathways. IGF-1 is a primary mediator of the action of growth hormone (GH) that modulates carbohydrate metabolism via insulin. Ageing results in reduced IGF-1 and GH levels [205], including in the brain [206]. AMPK is a sensor of cellular energy status, and its activation restores energy balance. Moreover, reduced AMPK activity is implicated in ageing [207]. mTOR, a serine-threonine protein kinase, is a negative regulator of ageing that promotes SASP [208]. In yeast, worms, and flies, blocking mTORC1 prolongs lifespan [209].

Nicotinamide adenine dinucleotide (NAD +) is a coenzyme central to energy metabolism and an essential cofactor in cellular redox reactions and SIRT activities [211]. It influences DNA repair, chromatin remodelling, and senescence, and reduced NAD + levels are detected during ageing [212, 213]. SIRTs are a family of seven proteins that regulate cell/tissue survival and metabolism, and they possess many functions associated with ageing. This includes DNA repair and genome stability, senescence, and mitochondrial function, and they inhibit oxidative stress, inflammation, and apoptosis [214, 215]. SIRT-1, 2, 3 and 6 also increase lifespan in species ranging from fruit flies to mammals [216–218]. Expression of SIRT1 decreases during ageing, hence elevating expression of SIRTs may protect against age-related events [219].

Changes in metabolic pathways are associated with the heterogeneity and diverse clinical characteristics of ALS. mTOR inhibition in mutant SOD1^G93A^ transgenic mice hastens disease progression and increases motor neuron degeneration [220]. However, mTOR inhibition is protective in a transgenic mouse model involving neuron-specific wildtype TDP-43 overexpression [221]. IGF-1 overexpression in primary motor neurons is protective against glutamate-induced toxicity in ALS [222]. AMPK activation has been detected in motor neurons of ALS patients as well as in the spinal cord of SOD1^G93A^ mice [223]. Dysregulation of SIRT has been described in ALS [224–226] and SIRT1 sensitive lysine-136 acetylation drives LLPS and pathological aggregation of TDP-43 [227, 228]. SIRT-1 activation has been examined therapeutically using resveratrol, which initially displayed promising effects by improving motor impairment and extending lifespan in SOD1^G93A^ mice [229]. However, it failed in clinical trials [230].

### Integrative hallmarks and ALS

#### Impaired intercellular communication

Cells can communicate with each other by either direct physical interactions or by intermediates such as extracellular vesicles (EVs) that act as inter-cellular messengers. During normal ageing, there is a gradual decline in the quality of communication between cells, which impacts on several processes relevant to ALS. These are discussed in the sections below.

##### Senescence and intercellular communication

Senescent cells are metabolically active and can communicate with, and influence the behaviour of, neighbouring cells through paracrine signalling [231]. Senescence is also an important part of inter-cellular communication and ageing [232] via SASP [233]. As well as the secretion of pro-inflammatory molecules, senescent cells also communicate with other cells via membrane-bound intercellular bridges or ‘tunnelling nanotubes’, that facilitate direct physical connections between cells [234]. The role of senescence in ageing and ALS is described in the 'Integrative hallmarks and ALS' section.

##### Neuroinflammation and inter-cellular communication between glia and neurons

Within the CNS, neurons, astrocytes, microglia, and oligodendrocytes must normally communicate with each other and the surrounding environment to maintain homeostasis. Motor neuron health and viability relies on efficient communication with glial cells and skeletal muscles [235, 236].

ALS is a non-cell-autonomous disease, and extrinsic inter-cellular communication amongst motor neurons, microglia, oligodendrocytes, and astrocytes is implicated in pathophysiology. This occurs through alterations in trophic factor support to motor neurons, signalling factors that impact on glial cell receptors and changes in direct cell-to-cell interactions [236]. Intrathecal administration of CSF from ALS patients in mice reduces the expression of trophic factors BDNF, fibroblast growth factor 2 (FGF2), and IGF-1 [237]. Pro-inflammatory cytokines and apoptosis-triggering TNF-α and Fas ligand (FASL) produced by activated microglia and astrocytes induce damage to motor neurons [238, 239]. Mouse cortical neurons treated with iPSC-derived astrocytes from C9ORF72 patients show increased oxidative stress and neurotoxicity [182]. Degenerating and morphologically altered oligodendrocytes are dramatically increased in mutant SOD1^G93A^ mice and are surrounded by clustered activated microglia [240]. Astrocytes derived from post-mortem familial ALS (SOD 1^A4V^) and sporadic ALS patient brains are toxic to motor neurons, but this is alleviated by reducing *SOD1* expression in astrocytes [241]. In mutant SOD1^G93A^ mice model, senescent astrocytes display less support to motor neurons. Furthermore, IL-6 levels increase in astrocytes of SOD1^G93A^ rodent models which recruits immune cells to clear the senescent cells [242, 243].

##### Extracellular vesicles and intercellular communication

EVs are tiny membrane-bound structures, typically ranging in size from 50 to 1000 nm [244]. They contain both protein and nucleic acid and they are released by various cell types in both physiological and pathological conditions [244]. Extracellular RNAs (exRNAs) are important mediators of cell-to-cell communication that are secreted as either EVs or in a complex with RNA binding proteins (RBPs)[245]. Senescence-associated EVs are implicated in the DDR and SASP [246, 247]. The levels of EVs alter during senescence and ageing, although it is controversial whether they increase or decrease [248–250].

ExRNAs and EVs are also implicated in ALS pathogenesis. Some exRNAs, including mRNA, microRNA and circular RNA, are present in exosomes and as they are dysregulated in ALS they have been proposed as potential biomarkers [174, 175] [251] (RNA dysregulation in ALS is reviewed in more detail in the 'Defects in RNA dysfunction section). Transgenic SOD1^G93A^ mice release astrocyte-derived EVs containing mutant SOD1^G93A^ that transfer to spinal neurons and selectively trigger death [252]. Microvesicles isolated from ALS patients contain higher levels of pathological proteins (SOD1, TDP-43, FUS) compared to controls, unlike exosomes, despite the mean size for both EV types being larger in ALS than controls [253].

Misfolded proteins are known to transmit between cells in ALS and other neurodegenerative diseases, particularly SOD1 [254, 255]. Several studies have described ‘prion-like’ characteristics of misfolded SOD1, including its capacity to transfer between cells and cause the misfolding of wildtype SOD1 within cells [256] and in vivo [257]. The transmission of toxic aggregates via EVs is not well-understood [244]. Misfolded SOD1, whether wildtype or ALS-associated variants A4V, G93A, G127X, are secreted as EVs in NSC-34 and HEK cells [254]. Astrocytes and neurons constitute the primary sources of EVs in vivo containing misfolded SOD1 in spinal cords of both SOD1^G93A^ transgenic mice and SOD1-ALS patients [258]. Similarly, ‘prion- like’ behaviour for TDP-43 has been described in mice [259, 260]. Further studies have demonstrated both exosome-dependent and independent mechanisms are involved in TDP-43 inter-cellular transmission [261]. Similarly, C9ORF72 DPRs, poly-GA, poly-GP, poly-GR, and poly-PA transmit from cell-to-cell by exosome dependent and independent mechanisms [262].

#### Neuroinflammation and ageing

Inflammation increases significantly during normal ageing, both systemically and in the nervous system (neuroinflammation). Senescent cells also contribute to the persistent inflammatory environment via SASP [162], and their accumulation leads to sustained inflammation [263]. Inflammasomes, multimeric protein complexes that activate inflammatory caspase 1, are integral components of the innate immune system that become activated during the ageing process [264]. This includes the nucleotide oligomerization domain (NOD)-like receptor protein 3 (NLRP3) inflammasome [265].

Neuroinflammation plays a significant role in ageing of the CNS and associated pathological conditions [266]. During ageing, activated microglia and astrocytes display altered morphologies and produce pro-inflammatory cytokines, leading to neuroinflammation [267–269]. When activated, astrocytes can display either neurotoxic, pro-inflammatory (A1) or neuroprotective, anti-inflammatory (A2) phenotypes. Similarly, microglia display both inflammatory and anti-inflammatory states, M1 and M2, respectively. RNA sequencing of brain-derived astrocytes throughout the lifespan of mice [270, 271] revealed up-regulation of A1-phenotype genes associated with neuroinflammation [271], linking astrocytes to cognitive impairment during ageing. Mice are protected from age-related reactive astrogliosis in the absence of microglial proinflammatory cytokines, suggesting that microglia are responsible for initiating the neuroinflammation that occurs with ageing [271]. However, astrocytes exert detrimental effects on microglia during ageing, impairing their phagocytic capabilities, resulting in a prolonged pro-inflammatory state [272].

Neuroinflammation is well-described in both human ALS and animal models [273]. Infiltration of peripheral lymphocytes, natural killer (NK) cells and macrophages, along with activation of astrocytes and microglia and the excessive production of inflammatory cytokines, is present in both humans and mice [274]. Interestingly, transcriptomic analysis of spinal cords of SOD1^G93A^ mice revealed a significant overlap (90% shared transcripts) between gene expression patterns associated with normal ageing and ALS, particularly inflammation and immune system activation [219]. The NLRP3 inflammasome, along with expression of caspase-1, IL-1β, IL-18, and NFκB, is increased in the SOD1^G93A^ transgenic rat [275]. In astrocytes of the spinal cord from SOD1^G93A^ mice [276] and sporadic ALS patients, elevated levels of NLRP3, apoptosis-associated speck-like protein containing a caspase-1 recruitment domain (ASC), IL18, and active caspase 1, are present [277]. Microglia in mutant and wild-type SOD1^G93A^ and TDP-43^Q331K^ ALS mice express NLPR3, consistent with elevated expression of inflammasome components in vivo [265]. TDP-43 binds to CD14 receptors in microglia, macrophages, and monocytes, activating NFκB and stimulating the NLRP3 inflammasome [278]. In SOD1^G93A^ rats, progression of paralysis was linked to neuroinflammation and motor neuron toxicity via microglia [174]. There is evidence for both neuroprotective and neurotoxic effects of astrocytes and microglia in ALS (reviewed in Clarke et al. 2020) [279, 280].

#### Stem cell exhaustion

Stem cells have self-renewal and multi-differentiation capabilities and thus regenerate tissue growth during ageing. Neural stem cells (NSCs) are responsible for producing neurons during prenatal development and maintaining the nervous system throughout adult life [281, 282]. However, during ageing, the functionality and regenerative capacity of NSCs deteriorates. This exhaustion of stem cells can be induced experimentally by upregulation of DNA damage, altered DNA repair mechanisms, decreased regenerative ability, epigenetic alterations, increased genomic instability, altered protein homeostasis, dysfunctional mitochondria, and senescence [290]. Several studies have identified possible ways to improve stem cell function during ageing, such as by increasing the levels of transcription factor FOXO4, HSP70 [290], or alternatively by exposing young blood to aged animals through heterochronic parabiosis [288].

A meta-analysis of eleven studies demonstrated that isolating and transplanting NSCs from the CNS into the spinal cord of transgenic mutant SOD1^G93A^ mice slowed disease progression [281]. This was related to improvement of neurotrophic factor production, reduced neuroinflammation, and preservation of neuromuscular function [281]. Regenerating and renewing aged stem cells may be beneficial therapeutically in neurodegenerative diseases including ALS, although this has not been well-studied.

#### Dysbiosis

The gut microbiome is now recognised to play a critical role in health and well-being [3] including ageing [4, 5], and it is shaped by genetics, age, stress, illness, medication, diet, and the environment. However, the microbiome is dysregulated in many pathological conditions, which is known as 'dysbiosis’ [3]. Most gut microorganisms are bacteria, and they are implicated in metabolism, defence against pathogens, development of the immune system, and synthesis of vitamins, short-chain fatty acids and other metabolites [3]. Importantly, the microbiome interacts with the CNS via the gut-brain axis, the bidirectional network linking the enteric nervous system to the CNS [2].

The gut microbiome is established during childhood. Whilst it displays significant diversity among individuals, [3] during normal ageing, changes in the composition of gut microbiota and reduced species diversity are associated with frailty, cognitive function, depressive symptoms, and inflammatory processes [3]. Furthermore, mouse models of progeria and progeria patients with HGPS or Nestor-Guillermo progeria syndrome (NGPS) display dysbiosis, characterised by loss and gain of specific species [36]. Transplantation of faecal microbiota between wildtype mice and progeria mice confirm the existence of a strong link to healthspan/lifespan [37, 38] and in the maintenance of brain health and immunity during ageing [11]. Similarly, administration of gut microbiota metabolites improves age-related pathologies in mice [2, 3]. Collectively, these findings suggest that ageing is closely associated with dysbiosis.

Dysbiosis is also linked to neurodegeneration in ALS. Dysregulation of the gut microbiome correlates with disease severity in both mutant SOD1^G93A^ transgenic mice and human patients [283]. Sodium butyrate is a bacterial metabolite produced in the gut by *Butyrivibrio fibrisolvens*, and reduced levels of this organism were detected in the SOD1^G93A^ mouse [284]. Increased intestinal permeability to toxins was also detected [284] and treatment of SOD1^G93A^ mice with butyrate also delayed weight loss and improved survival [285], implying that interventions aimed at restoring the gut microbiome may extend lifespan and healthspan in ALS. Alterations in gut microbiota have also been detected in C9ORF72-mutant mice [286], and C9ORF72 itself was found to inhibit systemic and neural inflammatory responses induced by gut bacteria [286]. Together these studies imply that the gut microbiome contributes both to ageing and the pathogenesis of ALS.

#### Defects in RNA functions

Defects in RNA metabolism are not included as a hallmark of ageing [3], but it has been proposed they should be designated as one, given increasing evidence highlighting their importance to ageing [287]. Given that ageing cells lose their ability to maintain RNA metabolism [288], and dysfunctional RNA metabolism is strongly implicated in the pathophysiology of ALS [289], here we consider this as an ageing hallmark that is discussed in relation to ALS.

The RNA milieu within a cell consists of coding messenger RNAs (mRNAs) and non-coding RNAs (ncRNAs), both of which interact with RNA-binding proteins (RBPs) within ribonucleoprotein complexes (RNPs). RBPs play important roles in RNA metabolism, including alternative splicing of pre-mRNA, transport, and stability, which are fine-tuned by modulation of their own expression and that of other RBPs [290]. They are also involved in modulation of SG dynamics by interaction with cytoplasmic RNAs and other RBPs. Dysregulation of RBPs also induces metabolic dysfunction, ageing, and senescence [291]. 

The transcriptome of the ageing cell results in global changes in gene expression  with down-regulation of genes related to oxidative respiration, protein translation and growth signalling, and up-regulation of genes related to innate immunity, inflammation and DNA damage [292–296]. The multiple layers of processing that determine gene expression, including mRNA modification such as splicing, capping and polyadenylation, RNA export, localization, turnover, and translation, are affected by ageing and ALS [297]. In multiple species, including humans and mice, ageing results in shorter RNA transcripts in nearly 80% of tissues, disrupting the balance of long and short RNA transcripts [297]. 

The signalling pathways that control alternative splicing are some of the most dysregulated processes in normal ageing [292, 298] and senescence [287, 299]. Higher rates of alternative splicing, including back-splicing and circular RNA formation, reduced transcript quality, and mismatches with genome sequences are also detected during ageing [296]. Furthermore, during natural ageing, cryptic splice sites become revealed. These are sequences within introns that incorporate into the transcript during splicing, resulting in a premature stop codon and loss of function of the associated protein [300].

The ageing transcriptome may further be influenced by altered RNA polymerase II (Pol II) activity [296, 301]. The speed of RNA polymerase II elongation within introns increases with age across multiple cellular and animal models and human samples [296]. In contrast, stalling of Pol II at DNA damage sites increases with age which results in transcriptional stress and shorter transcripts [301]. Cells have stringent RNA quality control systems to prevent these detrimental processes. Aberrantly spliced mRNA with premature stop codons are degraded by 'nonsense mediated decay (NMD)' in order to prevent translation into deleterious non-functional proteins [302]. This process is however dysregulated with ageing and it particularly affects post-mitotic neurons that are more dependent on strong RNA quality control and efficient NMD processes [302].

Chemical modifications to RNA regulate RNA metabolism and are known to contribute to at least eight of the classical hallmarks of ageing, including cellular senescence, epigenetic changes, immune and stem cell dysfunction, concomitant metabolic dysregulation and loss of proteostasis [303]. These RNA modifications include methylation and A-to-I editing. Decreased m^6^A modifications are present in aged human PBMCs [304], impairing synaptic protein synthesis and synaptic functions relevant to ageing and neurodegeneration [305], suggesting that m^6^A RNA methylation contributes to cognitive decline in ageing [305]. A-to-I editing also declines during ageing specifically in the human brain [306]. Similarly, mice lacking mRNA editing apolipoprotein B mRNA-editing enzyme catalytic polypeptide (APOBEC1) in microglia show acceleration of age-related neurodegeneration and motor deficiencies [307].

The expression of various ncRNAs is altered with ageing and influences its hallmarks. An array of ncRNAs, including long ncRNAs (lncRNAs), microRNAs (miRNAs), piwi-interacting RNAs (piRNAs), small nucleolar RNA (snoRNA), small nuclear RNA (snRNA), ribosomal RNA (rRNA), small Cajal body specific RNA (scaRNA), transfer RNA (tRNA) and tRNA derived fragments (tRFs), are differentially expressed in ageing tissues [308]. Of these, miRNAs were the most modified as a result of ageing [308]. Age-related changes in global gene expression also correlate with the corresponding miRNA expression [309], which is not surprising given that miRNAs regulate mRNA expression. LncRNAs regulate histone methyltransferases and other chromatin modifying enzymes and thus they epigenetically modify gene expression [310]. They may also protect cells from senescence, because the lncRNA senescence-associated noncoding RNA (SAN) was increased in aged adipose-derived stem cells (ASCs)[311] and another lncRNA, NEAT1 suppressed cellular senescence in hepatocellular carcinoma [312]. NEAT1 plays a crucial role in forming a flexible environment within cells, increasing LLPS and condensation of RBPs and nucleic acids [313]. CircRNAs, a more recently described class of mostly ncRNAs, are gaining recognition as potent regulators of gene expression via their interaction with miRNAs [314], and are emerging players in ageing [315] and age-related diseases [316], including ALS [317, 318].

There is now strong evidence that dysfunctional RNA metabolism is present in ALS. Interestingly, degenerating neurons in ALS show similar RNA metabolic defects (RNA processing, modifications and transport) to ageing neurons [298]. Some of the major proteins dysregulated in ALS are RBPs, including TDP43, FUS, TAF15 and hnRNPA1 [28, 291]. TDP43 and FUS mislocalise and aggregate in the cytoplasm in sporadic ALS [319], which reduces their expression in the nucleus [288], resulting in loss of essential functions, including splicing and regulation of transcription. Loss of nuclear TDP43 also leads to the emergence of cryptic splice sites, which are now increasingly recognised as contributors to ALS. This includes a cryptic splice site in the first intron of the stathmin-2 gene (STMN2), leading to loss of protein and inability to repair axons following motor neuron injury [320].

RNA modifications and quality control of RNA such as m^6^A methylation, A-to-I editing, NMD and RNA surveillance are also dysregulated in ALS [321–323]. The C9ORF72 expansion repeats sequester RNA export factors [324, 325] and inhibit NMD [326]. In contrast, NMD hyperactivation was detected in fibroblasts derived from ALS-associated FUS patients [327], which reduces protein biosynthesis and contributes to motor neuron death in ALS [328]. TDP-43 interacts with NEAT1 resulting in its condensation into nuclear bodies in response to stress and pathological features of ALS, such as phosphorylation and its mis-localisation [329].

These studies therefore provide significant evidence linking dysregulated RNA metabolism to ageing and ALS. However it is important to recognise that these events are intricately interconnected [330]. For example, loss of the RBP HuR causes a reduction in methylation (C106) of the lncRNA component of telomerase (TERC), impairing telomerase function and resulting in telomere attrition and accelerated ageing [331] (Table 1).

**Table 1:** Hallmarks of ageing in ALS

Hallmarks of ageing	Cellular pathways implicated in ALS	Pathological protein involved	References
Primary hallmarks			
Genomic Instability	Nuclear architecture alterations, nuclear pore pathology, damage to nuclear DNA, damage to mitochondrial DNA	SOD1, TDP-43, C9ORF72 DPRs, FUS, NEK1	[47, 48, 58, 60, 63, 64, 332, 333]
Telomere attrition	Both shorter and longer telomeres are described in ALS	SOD1, C9ORF72 DPRs	[77, 334, 335]
Epigenetic alterations	DNA hyper- and hypo-methylation, hyper- or hypo-acetylation of histones	SOD1, FUS, TDP-43, C9ORF72 DPRs	[88, 95–97, 100, 228, 336]
Loss of proteostasis	Defects in protein folding, disrupted UPS, defective ER-Golgi trafficking, ER stress, Golgi fragmentation and defects in ERAD	SOD1, TDP-43, C9ORF72 DPRs, FUS	[102, 103, 337–340]
Dysregulated macroautophagy	Increased or decreased activation of autophagy, impaired mitophagy, dysregulated autophagy initiation and impaired autophagic flux	C9ORF72 DPRs, SOD1, TDP-43, TBK1, FUS, FIG4, OPTN, UBLN2, SQSTM1, CHMP2B, ALS2	[341–348]
Antagonistic hallmarks			
Cellular senescence	Microglia senescence, abnormal expression of senescence markers	SOD1, C9ORF72	[174, 285, 349]
Mitochondrial dysfunction	Defects in membrane potential, decreased respiratory capacity, increased free radical production, reduced turnover, and dynamics	SOD1, TDP-43, C9ORF72 DPRs, UBQLN2 and FUS	[74, 191, 196, 332, 350–354]
Dysregulated nutrient sensing	Dysregulated mTOR signaling, AMPK pathway and SIRT regulation	SOD1, C9ORF72 DPRs, TDP-43	[210, 220, 355, 356]
Integrative hallmarks			
Impaired intercellular communication	Dysregulated interaction between glia and neurons, dysregulated EV and intracellular communication	SOD1, TDP-43, FUS and C9ORF72 DPRs	[244, 253, 357, 358]
Neuroinflammation	Hyperactivated astrocytes and microglia, increased pro-inflammatory cytokines Changes in glia to proinflammatory phenotypes	SOD1, TDP-43	[359–361]
Stem cell exhaustion	Decreased functionality and regenerative capacity of NSCs	SOD1	[362]
Dysbiosis	Increased intestinal permeability to toxins, altered gut microbiota	SOD1, C9ORF72 DPRs	[283, 284, 286]
Defects in RNA dysfunction	RNA metabolic defects (RNA processing, modifications and transport), splicing defects, cryptic exon inclusion, dysregulated quality control of RNA such as NMD and RNA surveillance	TDP43, FUS, TAF15 and hnRNPA1	[62, 317, 363–366]

## Ageing of motor neurons and non-neuronal cells in ALS

The hallmarks described above detail ageing-related events at the molecular and cellular level. Below, we also briefly discuss below how ageing specifically affects the cell types relevant to ALS; motor neurons, glia, and skeletal muscle cells.

### Ageing of motor neurons and glial cells in ALS

#### Motor neurons

It is unclear whether motor neurons are lost during normal ageing because conflicting findings have been obtained. Some studies have concluded that the size and number of motor neurons remains constant [367], whereas others report that there is a progressive motor neuron loss during physiological ageing [105, 368], similar to ALS, leaving the remaining aged motor neurons under stress [369].

It is clear that during normal ageing there are alterations in the properties of spinal α-motor neurons and a decline in neurotransmitter function [370]. This impairs their membrane and electrical properties, rendering them more susceptible to degeneration. Voluntary movements require efficient intra-neuronal excitatory (glutamatergic and cholinergic) and inhibitory (GABAergic and glycinergic) signalling [105]. Decreased cholinergic and glutamatergic synaptic inputs terminating on motor neurons are present in the ventral horn of old rhesus monkeys and mice [105]. In mice, membrane depolarization and increased expression of voltage-gated sodium channel isoform Na_v_1.8 l are present in aged motor neuron axons [371]. In addition, as neurons age, they lose their excitatory synaptic connections across the cell body and dendritic branches. Consequently, older motor neurons possess a diminished balance of excitatory to inhibitory synapses, which could impair their ability to initiate motor movements [305]. The expression of matrix metalloproteinase 1 (dMMP1) rises during ageing, which leads to motor functional impairments that worsen with ageing *Drosophila* motor neurons [372].

Alterations in synaptic transmission and the excitability of motor neurons are one of the first events in ALS. Hyperexcitability of both upper and lower motor neurons is frequently observed in SOD1^G93A^ mouse models, ALS-iPSC derived motor neurons, and ALS patients [373–375]. Excitotoxicity, referring to excessive activation of glutamate receptors and subsequent neuronal injury or death, is also commonly described in disease models [376]. During disease progression, however, motor neurons become hypo-excitable, although this could be a compensatory process [376]. In ALS patients, electrophysiological studies have identified abnormalities in sodium and potassium currents, implying that membrane depolarization and age-related changes in membrane excitability are present in median motor axons [377]. Increased expression of *Drosophila* dMMP1 in motor neurons contributes to the decline in motor function observed during ageing [372]. TDP-43 overexpression in neurons accelerates neuronal death by triggering dMMP1 expression, suggesting potential connections between ageing and ALS [372]. With ageing, motor neurons may become less efficient in transmitting signals to muscles, leading to slower response times and decreased motor control [378].

It remains unclear why motor neurons are selectively targeted in ALS. Neurons themselves display features that may render them more susceptible to the effects of ageing. Neurons rely on error-prone NHEJ for DSB repair [379], and being post-mitotic, they are unable to dilute the effect of DNA repair errors by cell division, unlike other cell types. Thus, they may be particularly vulnerable to DNA damage, as well as senescence [181, 379]. Post-mitotic neurons also may be more susceptible to the accumulation of misfolded proteins than other cell types, where the effect of protein aggregation can also be reduced by cell division. Hence, they are likely to be more susceptible to proteostasis dysfunction.

However, motor neurons possess distinctive characteristics compared to other neurons, which may render them uniquely vulnerable to neurodegeneration in ALS [1]. Motor neurons are large cells, with very long axons (up to 1m in an adult human), which may render them more prone to injury [380]. Also, they need to transmit signals over long distances and sustain constant firing and communication with muscles. Thus, the electrical activity of motor neurons may also contribute to their susceptibility in ALS, because this necessitates a significant amount of energy [380]. The constant firing of action potentials and the high metabolic rates required to maintain electrochemical gradients across neuronal membranes also increases oxidative stress [381]. Motor neurons are also highly susceptible to glutamate excitotoxicity compared to other neurons [382].

Within motor neurons, susceptibility to neurodegeneration in ALS is not uniform. Specific populations of motor neurons, including those in the oculomotor and Onuf's nuclei, remain relatively spared and do not degenerate until later stages of disease in humans [383] and mouse models [384–387]. Similarly, oculomotor neurons are not usually affected during ageing. In contrast, spinal motor neurons are targeted in both ALS and ageing, implying that ageing increases the susceptibility of spinal motor neurons to degeneration [388, 389]. Several studies have identified different gene expression profiles between oculomotor and spinal motor neurons. Microarray and laser capture microdissection of motor neurons isolated from oculomotor/trochlear nuclei, the hypoglossal nucleus, and the lateral column of the cervical spinal cord in humans and SOD1^G93A^ rats have revealed unique expression patterns in pathways associated with the ageing hallmarks, including loss of proteostasis, mitochondrial impairment and dysregulated autophagy [390, 391].

Differential susceptibility among motor neuron subtypes, even within the same motor unit, is also observed in ALS. Fast-fatigable (FF) motor neurons degenerate early in disease course, and the fatigue-resistant (FR) types subsequently follow later. In contrast, the slow (S) MNs are resistant to degeneration and are retained, even up to late in disease course. The reasons for the selective vulnerability of motor neuron subtypes remain unclear. However, both FF and FR subtypes are affected first during normal ageing whereas the slow subtypes are affected later, implying that ageing increases the susceptibility of FF and FR motor neurons in ALS [392–394]. The motor neuron subtypes also display noticeable differences in their properties. FF motor neurons possess somas with large-diameter whereas S motor neurons contain much smaller soma [385]. Also, FF motor neurons are much less excitable than the FR subtype which in turn are less excitable than the S subtype, also linking motor neuron susceptibility to excitability. Different gene expression profiles are also evident between these subgroups [385].

The unique combination of features in motor neurons, including their long axons, high energy demands, increased DNA damage and error-prone DNA repair mechanisms, neuronal senescence, susceptibility to excitotoxicity, and heterogeneity in susceptibility among subtypes, may therefore collectively render them more vulnerable to the effects of ageing [395]. Further exploration of these aspects is crucial for understanding the mechanisms underlying age-related motor neuron degeneration. Characterising these events may pave the way for targeted interventions to promote motor neuronal health during the normal ageing process.

#### Glial cells

Age-related changes and the presence of the ageing hallmarks are also detected in glial cells, as detailed below. The role of neuroinflammation induced by reactive astrocytes and microglia is discussed in the 'Neuroinflammation and ageing' section.

##### Astrocytes

Ageing is associated with morphological changes to astrocytes, characterized by atrophy and shrinking of processes such as branches and leaflets, and a decline in their function [396]. More specifically during ageing there is a decrease in synaptic connections and plasticity [397]. Astrocyte marker glial fibrillary acidic protein (GFAP) is highly increased in the aged brain, representing activation and gliosis during neurodegeneration [398]. The blood brain barrier is also maintained by astrocytes which also becomes compromised and leaky during ageing [398]. Dysregulation of astrocyte function, leading to sustained release of pro-inflammatory molecules such as IL-8, IL-1β, IL-6, IL-18, TNF-α, is implicated in ageing and age-related neurogenerative diseases [399].

Many studies have implicated astrocytes in ALS pathogenesis. A meta-analysis of studies involving human iPSC-derived astrocytes with variations in *SOD1, C9ORF72,* and *FUS*, with those using mouse astrocyte models expressing the SOD1^G93A^ mutation or TDP-43 deletion, or Tmem259 (membralin) deletion, revealed a consistent pattern of gene expression changes amongst these models [400], involving upregulation of genes associated with extracellular matrix dynamics, ER stress responses, and the immune system [400]. Reactive astrocytes induce neurofilament and SOD1 aggregation by disrupting autophagy via the action of TGF-β1, leading to motor neuron degeneration [401]. This occurs through the disruption of autophagy, primarily mediated by TGF-β1 [401]. Increased oxidative stress, reduced survival of motor neurons and neurotoxicity were observed in human iPSC-derived astrocytes from C9ORF72 ALS patients [182]. Similarly, iPSC-derived astrocytes from FUS ^R521H, P525L^ ALS patients impair motor neuron-neurite outgrowth, and formation and functionality of the NMJ formation [402].

##### Microglia

Microglia also undergo morphological changes with age [29]. In aged rats, microglia are characterized by shortened and less intricately branched structures containing nerve fibres and myelin sheets [403]. In the brains of aged individuals, a subset of microglia demonstrated elevated expression of activation markers, including MHC classII, CD45, and CD4. This expression pattern suggested that aged microglia exhibited increased inclusions, indicative of heightened phagocytosis activity [404]. Aged microglia cells also display altered mTOR signaling and increased oxidative stress [29].

There is debate whether microglial activation is present in ALS patients—transitioning from a ramified or stellate shape (inactive state) to an ameboid form (active state)—and whether there is increased proliferation and/or upregulation of inflammatory pathways in post-mortem ALS tissue [279, 405]. Evidence from mouse models including SOD1^G93A, A4V^, TDP-43^WT, M337V, A315T^, FUS^WT^ and C9ORF72 knockouts revealed microglia activation and morphological changes compared to controls [279, 406–408]. Reactive microglia are neuroprotective in a TDP-43 mice model [409]. Monocyte-derived microglia-like cells display pathological hallmarks of ALS, including cytoplasmic aggregation and phosphorylation of TDP-43, DNA damage, and cell-specific impairment of phagocytosis associated with disease progression [410].

##### Oligodendrocytes

Whilst oligodendrocytes normally myelinate axons in the CNS [411], during ageing, myelin regeneration becomes slowed [411]. Ageing oligodendrocytes also display reduced expression of myelin-associated genes *MOG, PLP*, and *CNP* [412], and *HMGCS1*, which is associated with cholesterol synthesis [412]. Conversely, ageing oligodendrocytes display increased expression of genes related to ribosome biogenesis*, RPl6, RPS29, and RPl23A*, and upregulation of immune-related genes such as *C4B and Il33* [412].

Selective removal of mutant SOD1 selectively from oligodendrocytes substantially delays disease onset and prolonged survival in SOD1^G93A^ mice [413]. Oligodendrocytes supply energy to axons through glucose and lactose shuttling and blockage in  these pathways  is implicated in motor neuron degeneration in ALS [414]. Oligodendrocytes also induce motor neuron death via human SOD1-dependent mechanisms in ALS [415]. Morphological changes and the presence of TDP-43 inclusions were detected in oligodendrocytes in human sporadic ALS spinal cords [416].

##### Schwann cells

Schwann cells are peripheral myelin generating cells that have a remarkable capacity to regenerate and remyelinate but during ageing these abilities decline [417, 418]. The ageing process in Schwann cells is also linked to abnormalities in myelination in mice [418]. Moreover, older mice show a significant decrease in the number of myelinated nerve fibres [419]. 

The role of Schwann cells in ALS pathology is poorly understood. However, they induce peripheral nerve inflammation through expression of CSF1, IL-34, and SCF factors in ALS patients [420, 421] and transgenic SOD1^G93A^ rodents [421, 422]. Reducing the levels of SOD1^G37R^ from Schwann cells accelerated disease progression in  mice [384]. Terminal and pre-terminal Schwann cells are lost in transgenic SOD1^G93A^ mice, which impairs reinnervation following muscle denervation [422].

### Ageing of skeletal muscle/NMJs

Physiological ageing can significantly affect skeletal muscles [423], both structurally and functionally. This can result in progressive loss of muscle mass or ‘sarcopenia’, leading to muscle weakness and motor control [423, 424]. The neuromuscular junction (NMJ) is a specialized tripartite chemical synapse that entails highly coordinated communication between the presynaptic motor neuron, postsynaptic skeletal muscle, and terminal Schwann cells. Muscle denervation is a major contributor to sarcopenia [425] and alterations in the NMJ during ageing play a pivotal role in this process [425–427]. In muscles, age-related decline in mitochondrial function [428] of 25–30% between the ages 30 and 70 years occurs [429]. Similarly, reduced oxygen consumption rates and elevated ROS production in muscles are present during normal ageing in mice [430].

Satellite cells display many classic hallmarks of ageing, and their capability to regenerate becomes compromised during normal ageing [185]. These are also similar to features detected in ALS, including increased DNA damage, mitochondrial dysfunction, loss of proteostasis, oxidative stress, and autophagy defects [351, 431]. Elderly satellite cells display disrupted antioxidant activity and increased membrane fluidity compared to those of young individuals [432], indicating age-dependent imbalance in the antioxidant system during ageing. Age related dysfunction of satellite cells and decreased regenerative capacity are also present in ALS [433].

Impaired NMJs are well-described in ALS although there is controversy whether this involves ‘dying forward’ (originating in the motor cortex before descending to the NMJ) or "dying back" (beginning at the NMJ and then propagating to the motor neuron cell body) mechanisms. Impairment in NMJs is detected in ALS patient tissues and disease models [434]. Transgenic SOD1^G93A^ mice [435] and mutant FUS^R521C^ mouse models exhibit changes in the NMJ early in disease course [387, 436]. Similarly, transgenic SOD1^G93A^ zebrafish display early defects in motor neuron outgrowth, axonal branching and dysregulated NMJ with reduced postsynaptic volume [437, 438]. ALS transgenic mice displaying TDP-43 pathology that lack the nuclear localization signal, also exhibit substantial muscle denervation [439]. Similarly, inducible expression of the C9ORF72 hexanucleotide repeat expansion in mice result in structural abnormalities in NMJs. In addition, early inhibition of C9ORF72 expression leads to structural NMJ abnormalities and rapid muscle dystrophy [440]. It has been proposed that as NMJ impairment in ALS leads to motor neuron dysfunction [441], and degeneration of motor neurons results in further NMJ dysfunction, this creates a viscous cycle, resulting in the progressive loss of muscle control and strength [441].

## Therapeutic interventions for ageing and ageing-related diseases

Whilst normal ageing is an inevitable consequence for all individuals, despite the increasing aged population, few therapeutic approaches have been developed for ageing itself. In contrast, there has been intense efforts to develop treatments for age-related diseases, including ALS [442]. There has been considerable progress in this area and there are now seven FDA-approved drugs for ALS. However, these treatments have limited efficacy or are restricted to specific groups of patients. Thus it is important to continue to develop new approaches.

During normal ageing there is a progressive decrease in functionality and in ALS this becomes accelerated. Thus, therapeutic approaches should aim to promote healthspan as well as lifespan. In humans, lifestyle interventions such as calorie restriction and exercise have been examined as approaches to prevent/delay ageing. Furthermore, numerous therapeutic studies have been performed in animal models, demonstrating that delayed ageing and prolonging longevity and healthspan are possible [442]. In particular, treatments that inhibit mTOR are showing promise [209]. In cells, senescence phenotypes such as size and granularity, β-galactosidase staining and fibroblastic spindle morphology are reversed by mTOR inhibition [443]. Rapamycin, an mTOR inhibitor, extends the lifespan of yeast, nematodes, fruit flies and mice [444]. Rapamycin is safe and well-tolerated in a randomised, double-blinded, placebo controlled ALS clinical trial, but further trials are necessary to understand the clinical and biological effects of the drug in ALS [445].

As the levels of NAD^+^ decline with normal ageing, NAD^+^ precursors that increase the levels of NAD^+^ have been examined therapeutically, including nicotinamide riboside (NR) and nicotinamide mononucleotide (NMN), NAD^+^ biosynthetic enzymes, and NAD^+^ degradation inhibitors [446]. NMN (CD38 inhibitor) and NR (PARP inhibitor) administration in transgenic SOD1^G93A^ mice delay senescence, improve stem cell renewal, and enhance lifespan [447]. However, deletion of CD38 had no effect in survival in two hSOD1 ALS mouse models [448, 449]. These compounds are in various stages of human clinical trials for ALS [335, 449].

Compounds that enhance SIRT activity may also be protective against ageing given that this function is decreased with age [450]. These include polyphenols such as resveratrol, which activates SIRT1 NAD^+^-dependent histone deacetylase activity [451]. Administration of dietary resveratrol extends lifespan of several organisms including yeast, nematode and fruit flies [452] and mice models of ageing [453]. Resveratrol also enhances mitochondrial biogenesis and improves oxidative capacity in ageing mice models [454]. Resveratrol treatment also prevented motor neuron loss, relieved muscle atrophy, and improved mitochondrial function in muscles [455] and extended survival in SOD1^G93A^ mice [229]. However, another study reported that  in SOD1^G93A^ mice it did not improve motor function or increase survival when given as a single dose, in contrast to these other studies [456].

Metformin, used in Europe as traditional medicine since the early nineteenth century, is also implicated as an anti-ageing treatment, because it suppresses cellular senescence phenotypes [457] and has been described as a possible protective factor against neurodegeneration [458, 459]. Metformin treatment of iPSCs generated from HGPS patients decreases progerin expression and reduces abnormalities in nuclear architecture, suggesting it has therapeutic potential to reduce ageing [460]. Lonafarnib is a farnesyltransferase inhibitor that reduces the effects of progerin on nuclear morphology in HGPS and is the only FDA approved treatment for HGPS [461]. FOXO4 peptide treatment and immunotherapy reverses senescence-associated loss of tissue by restoring the apoptotic role of p53 [462]. A small molecule telomerase activator, TA-65, inhibited telomere shortening and enhanced immune function [463], increased healthspan and rescued DNA damage defects in old adult mice [464]. Similarly, supplementation of TA-65 led to an improvement of healthspan indicators in several studies [465]. A recent RNA-seq analyses identified IGF1 and mitochondrial translation as longevity signatures common to 41 mammalian species and identified compounds that extended lifespan in mice [466]. However, none of these approaches have yet been tested in ALS (Table 2).

**Table 2:** Therapeutic compounds targeting the ageing hallmarks

Treatment	Properties/ Mechanism of action	Trial/ Study	Result	References
Vitamin E	Antioxidant	Randomized placebo-controlled human clinical trial (RCT)	No significant improvement in disease progression or survival	[467, 468]
Vitamin C and Carotenoids Supplementation	Antioxidant	Pooled results from 5 different cohort studies	Did not significantly reduce the risk of developing ALS	[469]
EH301, (PT and NR)	Combination of antioxidant and anti-aging agent	Pilot RCT	Slowed disease progression in an ALS patient	[470]
Rapamycin	mTOR inhibitor	Phase 1/II RCT	Treatment was safe and well-tolerated. Efficacy studies are required	[445]
NMN	CD38 inhibitor	Pre-clinical SOD1^G93A^ mice study	Treatment delayed senescence, improved stem cell renewal, and enhanced lifespan	[448, 471]
NR	PARP inhibitor	Pre-clinical SOD1^G93A^ mice study	Treatment delayed senescence, improved stem cell renewal, and enhanced lifespan	[471]
Resveratrol	SIRT 1 activation	Pre-clinical SOD1^G93A^ mice study	Treatment prevented motor neuron loss, relieved muscle atrophy, and improved mitochondrial function in muscles and extended survival in SOD1^G93A^ mice	[229, 230, 455]
Trichostatin A, scriptaid, tubastatin A	HDAC inhibitors	Pre-clinical SOD1^G93A^ mice study	Improved lifespan, delayed disease onset and improved motor function in the SOD1^G93A^ model	[85, 472]
Valproic acid, entinostat, sodium phenylbutyrate	HDAC inhibitors	Pre-clinical SOD1^G93A^ mice study	Improved lifespan, delayed disease onset and improved motor function in the SOD1^G93A^ model	[85, 472]
Sodium phenylbutyrate and valproic acid	HDAC inhibitors	Phase II RCT	Safe and tolerable	[85, 472]

DNMTs, HDACs and HATs have also been examined in ageing. HDACs include the ‘classical’ Zn^2+^-dependent deacetylases (class I, II, and IV) and SIRT deacetylases (class III) that are also implicated in longevity [473]. Inactivating HDAC homologs in *C*. *elegans* and *Drosophila* improved lifespan, and delayed age-related physical decline [336]. HDAC inhibitors trichostatin A, scriptaid, tubastatin A, valproic acid, entinostat, sodium phenylbutyrate improved lifespan, delayed disease onset and improved motor function in SOD1^G93A^ mice [101, 472]. HDAC inhibitors may therefore have potential in age-related diseases such as ALS [474]. However, whilst HDAC inhibitors  sodium phenylbutyrate and valproic acid are safe and tolerable in human ALS phase II clinical trials, there was no difference in survival compared to placebo [101].Therapeutic strategies involving preventing protein aggregation and restoring proteostasis have been widely studied in ALS. Molecular chaperones such as HSPs [475] and PDI family members are neuroprotective against ALS mutant SOD1 and TDP-43-induced pathologies, including in vivo [115, 338, 476]. Compounds targeting neuroinflammation have largely been ineffective in ALS clinical trials, although a phase II study involving tyrosine kinase inhibitor masitinib with riluzole showed promising results in ALS patients [477].

## Conclusion

The universal phenomenon of ageing is the largest risk factor for age-related neurodegenerative diseases, including ALS [335, 478]. Hence, therapeutic strategies that target ageing mechanisms may be beneficial in these conditions. However, to date, ageing has not been explored specifically as a drug development target in relation to ALS. Furthermore, the molecular links between ageing and neurodegeneration remain poorly understood. Normal ageing is a complex, multi-layered phenomenon that is difficult to separate from many age-associated diseases. Furthermore, the recognised molecular and cellular hallmarks of ageing overlap significantly and are strongly related to each other (Fig. 1**)**. Moreover, there are numerous similarities between ageing and the pathophysiology of ALS. However, whilst most neurodegenerative mechanisms implicated in ALS are related to the key hallmarks of ageing, more studies are required to confirm a direct connection between dysfunction in these events and ageing in ALS. The pathophysiology underlying ALS involves a complex interaction between ageing, genetic, epigenetic, and environmental factors. As pathogenesis in ALS is thought to be multistep process requiring six steps [479], ageing may therefore be an important accelerator of neurodegeneration in ALS.

Given the complexity of ALS, it is important to employ a multifaceted approach to target age-associated molecular mechanisms. Maintenance of the integrity of both the genome and proteome is of central importance in this process and increasingly described links between these pathways are described [479, 480]. Similarly, restoring proteostasis and improving resilience to cellular stress will be essential to withstand the insults associated with ALS. The increasing recognition of skeletal muscle in the etiology of ALS must also be considered, particularly as many features of normal ageing in muscle are also described in ALS. However, understanding the primary mechanisms that drive ageing at molecular and cellular level will facilitate in unravelling the mysteries of why the risk of ALS increases with age. Normal ageing is an inevitable consequence for all individuals but it will be important to consider age-related molecular events when considering novel therapeutic approaches for ALS in the future to design more effective strategies to halt neurodegeneration [442].

## Data Availability

Not applicable.
